# Comparative Efficacy and Safety of First-Line Immune Checkpoint Inhibitors Plus Chemotherapy with or Without Bevacizumab in Advanced Non-Squamous Non-Small Cell Lung Carcinoma

**DOI:** 10.3390/curroncol33030173

**Published:** 2026-03-18

**Authors:** Ping Chen, Mengchi Wang, Siyan Peng, Honglin Zhu, Yanming Wang, Zixuan Wan, Xuan Yang, Zhixin Yu, Yixin Zhou

**Affiliations:** 1 Department of VIP Region, Sun Yat-sen University Cancer Center, Guangzhou 510060, China; 2 Key Laboratory of Oncology in South China, Collaborative Innovation Center for Cancer Medicine, Guangdong Key Laboratory of Nasopharyngeal Carcinoma Diagnosis and Therapy, Guangzhou 510060, China; 3 Zhongshan School of Medicine, Sun Yat-sen University, Guangzhou 510060, China; 4 Department of Medical Oncology, Sun Yat-sen University Cancer Center, Guangzhou 510060, China; 5 Department of Experimental Research Cancer, Sun Yat-sen University Cancer Center, Guangzhou 510060, China

**Keywords:** non-small cell lung cancer, immunotherapy, bevacizumab, progression-free survival, propensity score matching, network meta-analysis

## Abstract

**Background:** First-line chemoimmunotherapy (I + C) is the standard of care for advanced non-squamous non-small cell lung cancer (NSCLC) without oncogenic mutation. Bevacizumab has been shown to enhance the efficacy of chemotherapy in non-squamous NSCLC, yet its added value when combined with I + C (I + C + B) remains unclear. To address this gap, we conducted a real-world comparative study and a network meta-analysis to evaluate I + C + B versus I + C in this setting. **Methods:** This retrospective study included patients with advanced EGFR/ALK-negative non-squamous NSCLC treated with first-line I + C + B or I + C. Propensity score matching (PSM) was employed to balance baseline characteristics between groups. Efficacy endpoints were progression-free survival (PFS) and overall survival (OS). Subgroup analyses examined outcomes by PD-L1 expression, age, metastases, and chemotherapy, among other factors. In parallel, a network meta-analysis of four randomized trials (*n* = 2026) indirectly compared I + C + B against I + C for PFS, OS, and safety outcomes. **Results:** A total of 277 patients were included, with 167 (60.3%) receiving I + C + B and 110 (39.7%) receiving I + C. Before PSM, the I + C + B regimen significantly prolonged PFS versus I + C (hazard ratio [HR] = 0.69, 95% CI 0.52–0.92, *p* = 0.010), with this benefit maintaining post-matching (HR = 0.70, 95% CI 0.49–0.99, *p* = 0.045). However, OS did not differ significantly between groups in either the pre-PSM (HR = 0.93, 95% CI: 0.67–1.30; *p* = 0.665) or matched analyses (HR = 0.84, 95% CI: 0.54–1.29; *p* = 0.421). Subgroup analyses suggested greater PFS benefit from I + C + B among PD-L1-negative, older patients, those with brain metastases or multiple metastatic sites, and in patients receiving specific chemotherapy doublets. The network meta-analysis confirmed a PFS advantage for I + C + B over I + C (HR = 0.84, 95% CI: 0.71–0.98) without an OS benefit (HR = 0.95, 95% CI: 0.79–1.14). Toxicity was higher with I + C + B; rates of grade 3–5 adverse events, serious adverse events, and treatment discontinuation were all significantly increased compared to I + C. **Conclusions:** In the first-line treatment of advanced EGFR/ALK-negative non-squamous NSCLC, adding bevacizumab to I + C improved PFS but did not translate into an OS gain. Although PFS benefits were observed in certain subgroups, these were accompanied by significantly increased treatment-related toxicities. Our findings suggest that no clear subgroup has been identified where the benefit outweighs the risks, necessitating extreme clinical caution.

## Simple Summary

For patients with advanced non-squamous non-small cell lung cancer lacking EGFR or ALK mutations, initial treatment typically involves a combination of chemotherapy and immunotherapy. While adding the anti-angiogenic drug bevacizumab to chemotherapy has previously shown benefit, its role when combined with modern chemoimmunotherapy was uncertain. This study found that incorporating bevacizumab into first-line chemoimmunotherapy significantly improved progression-free survival, especially for patients with high-risk features. However, this was accompanied by significantly increased treatment-related toxicities and no overall survival benefit. Consequently, our data identified no clinical subgroup where the benefit clearly outweighs these risks, necessitating extreme caution in its clinical application.

## 1. Introduction

Non-small cell lung carcinoma (NSCLC), accounting for approximately 85% of lung cancer cases, remains the leading cause of cancer-related mortality worldwide [1,2]. Among NSCLC subtypes, non-squamous NSCLC predominates and often harbors oncogenic driver mutations, such as EGFR or ALK alterations, for which targeted therapies have significantly improved patient outcomes [3,4,5]. Concurrently, notable advancements have also been achieved for patients lacking driver mutations.

Immune checkpoint inhibitors combined with chemotherapy (I + C) have been established as a standard first-line treatment for patients with driver gene-negative NSCLC, significantly reshaping the therapeutic landscape [6,7,8]. The KEYNOTE-189 trial demonstrated that pembrolizumab combined with pemetrexed–platinum chemotherapy significantly improved progression-free survival (PFS) to 9.0 months (hazard ratio [HR] = 0.50) and yielded an objective response rate (ORR) of 48.3% [9,10]. Similarly, the IMpower130 study reported a median PFS of 7.0 months (HR = 0.64) with atezolizumab plus nab-paclitaxel and carboplatin [11]. Additional pivotal trials evaluating various I + C regimens have consistently shown median PFS ranging from 7.0 to 11.3 months and ORR from 47.6% to 60.5% [12,13,14]. Despite these promising results, therapeutic efficacy for driver gene-negative NSCLC appears to have reached a plateau, underscoring the necessity of further exploring strategies to enhance clinical outcomes.

Recent evidence from the phase III HARMONi-2 trial has reignited interest in the role of anti-angiogenic therapy in the first-line treatment of advanced NSCLC. In this study, the dual inhibition of PD-1 and VEGF demonstrated significantly improved progression-free survival compared to immune monotherapy in PD-L1-positive and EGFR/ALK-negative NSCLC [15], highlighting the potential synergistic effect of combining anti-angiogenesis and immunotherapy strategies. This renewed attention prompts a re-evaluation of earlier studies such as IMpower150, which investigated the efficacy of adding bevacizumab to an immune checkpoint inhibitor plus chemotherapy regimen (I + C + B). In patients without EGFR or ALK mutations, IMpower150 demonstrated that I + C + B significantly improved overall survival (OS) to 19.2 months compared to 14.7 months with bevacizumab plus chemotherapy (HR = 0.78; *p* = 0.02), and it improved median PFS to 8.3 months versus 6.8 months (HR = 0.62; *p* < 0.001) [16,17,18]. Nevertheless, the clinical benefit of incorporating bevacizumab into I + C regimens remains controversial. While IMpower150 showed encouraging results, other trials such as APPLE did not observe a significant improvement in either PFS or OS, raising concerns regarding the additional toxicity and cost in relation to benefit [19]. These inconsistencies highlight the necessity of further investigation to better define the value of I + C + B strategies.

To address this gap, we conducted a single-center retrospective study to compare the real-world efficacy of I + C + B against I + C regimens in advanced EGFR/ALK-negative non-squamous NSCLC. Additionally, we performed a network meta-analysis of existing randomized controlled trials evaluating these approaches. Our goal is to provide comprehensive, evidence-based insights into the efficacy and safety of the I + C + B combination in this patient population.

## 2. Methods

### 2.1. Inclusion Criteria and Data Collection of Real-World Cohort

This retrospective study was conducted at Sun Yat-sen University Cancer Center and included patients diagnosed with advanced lung cancer between May 2019 and September 2023, ensuring an adequate follow-up duration for mature data analysis (Figure S1). All patients were censored at the designated data cut-off.

Eligible participants met the following inclusion criteria: (1) age ≥ 18 years; (2) histologically or cytologically confirmed stage IV NSCLC without EGFR/ALK mutations; (3) receipt of at least two cycles of systemic therapy following diagnosis; (4) first-line treatment with either I + C or I + C + B; and (5) availability of complete clinical data. Collected variables included patient demographics, tumor histology, metastatic sites, treatment regimens, PD-L1 expression (tumor proportion score, TPS) (Table 1), radiological response, survival outcomes, and treatment-related adverse events (AEs).

PD-L1 expression was determined using the Dako 22C3 PharmDx assay (Dako, Carpinteria, CA, USA) and quantified via the TPS. For patients without baseline PD-L1 data, we retrospectively retrieved archived pre-treatment formalin-fixed paraffin-embedded tumor blocks and obtained fresh 4 μm sections for supplementary testing; for others, results were extracted from existing medical records.

### 2.2. Study Eligibility and Data Extraction of Meta-Analysis

A systematic literature review was conducted to identify clinical trials evaluating I + C or I + C + B as first-line therapy for patients with advanced non-squamous NSCLC lacking EGFR or ALK alterations. Eligible studies were retrieved from PubMed, Embase, and the Cochrane Central Register of Controlled Trials, and were further supplemented with screening abstracts from major oncology conferences including ESMO, ASCO, and WCLC (File S1; Figure S2). Only English-language publications available through 20 May 2024 were considered. Adhering to PRISMA-NMA guidelines, the completed checklist is provided in File S2.

Two investigators (Ping Chen and Mengchi Wang) independently extracted data using a standardized collection template. Discrepancies were resolved via consensus. Extracted information included trial acronym, study design, sample size, year of publication or presentation, patient characteristics, and key outcomes—including PFS, OS, and AEs among patients with EGFR/ALK wild-type non-squamous NSCLC. Risk of bias was assessed independently by two reviewers (Ping Chen and Mengchi Wang).

### 2.3. Endpoints and Statistical Analysis

The primary endpoint for the real-world cohort was PFS, defined as the interval from treatment initiation to the first occurrence of documented disease progression or death. Secondary endpoints included OS, ORR, and AEs. OS was defined as the time from treatment initiation to death from any cause. Tumor responses were evaluated in accordance with RECIST version 1.1 [20], classifying outcomes as complete response (CR), partial response (PR), stable disease (SD), or progressive disease (PD). Radiographic assessments were independently reviewed by two investigators, with discordant evaluations resolved through consensus discussion among the full study team. ORR was defined as the proportion of patients achieving either CR or PR. AEs were assessed and classified according to the National Cancer Institute Common Terminology Criteria for Adverse Events, version 5.0 (CTCAE v5.0). These data were independently assessed by two investigators (Ping Chen and Mengchi Wang), with any discrepancies resolved through consensus discussion.

To reduce selection bias and ensure comparability between the I + C + B and I + C treatment groups, propensity score matching (PSM) was employed. Propensity scores were estimated using a logistic regression model incorporating baseline variables with a univariate *p* value < 0.25, including age, presence of brain metastases, pleural or pericardial effusion, and chemotherapy regimen. One-to-one nearest-neighbor matching without replacement was performed using a caliper set at 0.05 standard deviations of the logit of the propensity score. Matching quality was evaluated using standardized mean differences (SMDs), with values < 0.10 considered indicative of adequate covariate balance between treatment arms [21].

Continuous variables were presented as mean ± SD or median (range), while categorical variables were reported as numbers (%). Survival outcomes were analyzed using the Kaplan–Meier method, and comparisons between groups were made with the log-rank test. Associations between categorical variables were assessed using the Chi-square test or Fisher’s exact test, as appropriate. Odds ratios (ORs) and risk ratios (RRs), along with their respective 95% confidence intervals (95% CIs), were calculated to compare objective response rates (ORRs) and adverse event rates, respectively, between the study groups. For subgroup analyses involving five or more patients, multivariate Cox proportional hazards models were constructed to estimate HRs and 95% CIs.

In the meta-analysis, the primary outcome was OS, with PFS and AEs as secondary endpoints. HRs and 95% CIs for OS and PFS were either directly extracted from included studies or derived from reported statistics when necessary. AE data were aggregated via treatment regimen and reported as pooled percentages.

Inter-study heterogeneity was assessed using the Cochrane Q test and quantified via the *I*^2^ statistic, which reflects the proportion of total variation due to heterogeneity rather than sampling error. A network meta-analysis framework was used to indirectly compare the efficacy of I + C + B versus I + C regimens. Node-splitting analysis was used to assess the agreement between direct and indirect evidence. Pooled HRs with 95% CIs for OS and PFS were calculated using the inverse variance method. For dichotomous AE data, pooled RRs and 95% CIs were computed using the Mantel–Haenszel method. Fixed-effects models were utilized as the number of included studies was small (*n* < 5) [22]. To provide a robust interpretation of treatment priority and avoid overstating ranking precision, a rank-cluster approach (RaCE model) was employed using the RaCE.NMA R package [23]. The risk of bias in the included trials was evaluated using the Cochrane Collaboration’s tool [24].

All statistical analyses were performed using SPSS version 26.0 (Version 31.0, IBM Corp., Armonk, NY, USA), R version 4.4.1 (R Foundation for Statistical Computing, Vienna, Austria), and Stata version 15.0 (StataCorp, College Station, TX, USA). A two-sided *p* value < 0.05 was considered statistically significant.

## 3. Results

### 3.1. Clinical Characteristics of the Real-World Cohort

A total of 277 patients with advanced non-squamous NSCLC lacking EGFR or ALK mutations were included in the primary analysis cohort (Figure S1). Baseline demographic and clinical characteristics are summarized in Table 1. Among the enrolled patients, 110 (39.7%) received I + C, while 167 (60.3%) received I + C + B as first-line therapy. Notably, the I + C + B group was generally younger and used more paclitaxel-based chemotherapy. PD-L1 status was available for 54.2% of patients (*n* = 150). In the I + C group, 21.8% were PD-L1-negative (<1%) and 36.4% were positive (≥1%); in the I + C + B group, 16.8% were negative and 34.7% were positive. PD-L1 distribution was well-balanced between groups (*p* = 0.450). For the entire cohort, the median follow-up time was 34.53 months (95% CI: 31.05–37.82), with a censoring proportion of 48.0%.

Following propensity score matching, 80 patients were retained in each treatment group. Post-matching comparisons indicated well-balanced baseline characteristics between the two arms, confirming the adequacy of the matching process.

### 3.2. Survival Analyses of the Real-World Cohort

**Figure 1:**
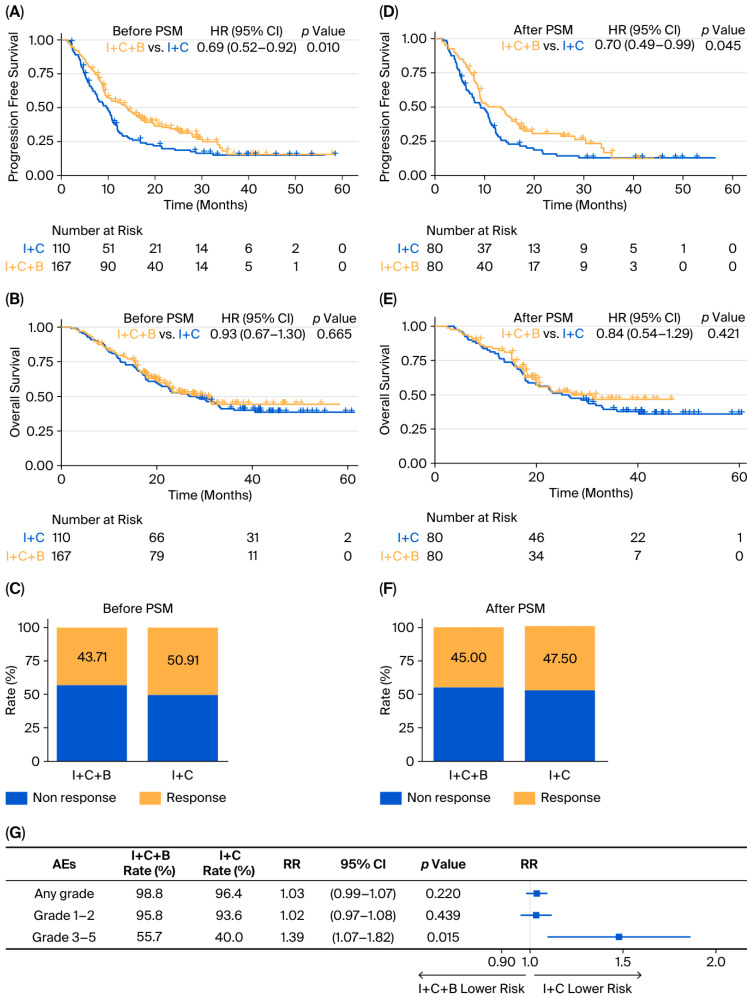
**Treatment efficacy and AEs comparisons between I + C + B and I + C regimens in the real-world cohort.** (**A**–**C**): PFS, OS and ORR in the unmatched cohort; (**D**–**F**): PFS, OS and ORR in the cohort post-PSM; (**G**) forest plot of AEs. Abbreviations: I + C + B, immune checkpoint inhibitors plus chemotherapy and bevacizumab; I + C, immune checkpoint inhibitors combined with chemotherapy; PSM, propensity score matching; HR, hazard ratio; CI, confidence interval; AEs, adverse events; RR, risk ratio.

In the unmatched cohort, the median PFS was 14.03 months (95% CI: 10.28–17.02) in the I + C + B group versus 9.71 months (95% CI: 7.92–10.97) in the I + C group, corresponding to an HR of 0.69 (95% CI: 0.52–0.92; *p* = 0.010) (Figure 1A). Median OS was 29.11 months (95% CI: 22.44–not reached) in the I + C + B arm and 27.56 months (95% CI: 20.57–40.54) in the I + C arm (HR = 0.93; 95% CI: 0.67–1.30; *p* = 0.665) (Figure 1B). The ORR before matching was 43.71% in the I + C + B group and 50.91% in the I + C group (OR = 0.75; 95% CI: 0.46–1.21; *p* = 0.241) (Figure 1C).

After PSM, the median PFS was 13.30 months (95% CI: 8.97–16.66) for the I + C + B group and 8.94 months (95% CI: 6.74–11.20) for the I + C group (HR = 0.70; 95% CI: 0.49–0.99; *p* = 0.045) (Figure 1D). The 1-year and 3-year PFS rates were 50.2% and 12.5% in the I + C + B group, compared with 31.0% (*p* = 0.013) and 12.9% (*p* = 0.958) in the I + C group. The corresponding median OS was 27.24 months (95% CI: 20.21–not reached) and 25.76 months (95% CI: 18.20–40.54), respectively (HR = 0.84; 95% CI: 0.54–1.29; *p* = 0.421) (Figure 1E). The 1-year and 3-year OS rates were 83.6% and 46.8% for the I + C + B group, and 80.00% (*p* = 0.559) and 39.4% (*p* = 0.390) for the I + C group. Post-PSM ORR was 45.00% and 47.50%, respectively (OR = 0.904; 95% CI: 0.486–1.684; *p* = 0.751) (Figure 1F).

### 3.3. Subgroup Analyses of the Real-World Cohort

**Figure 2:**
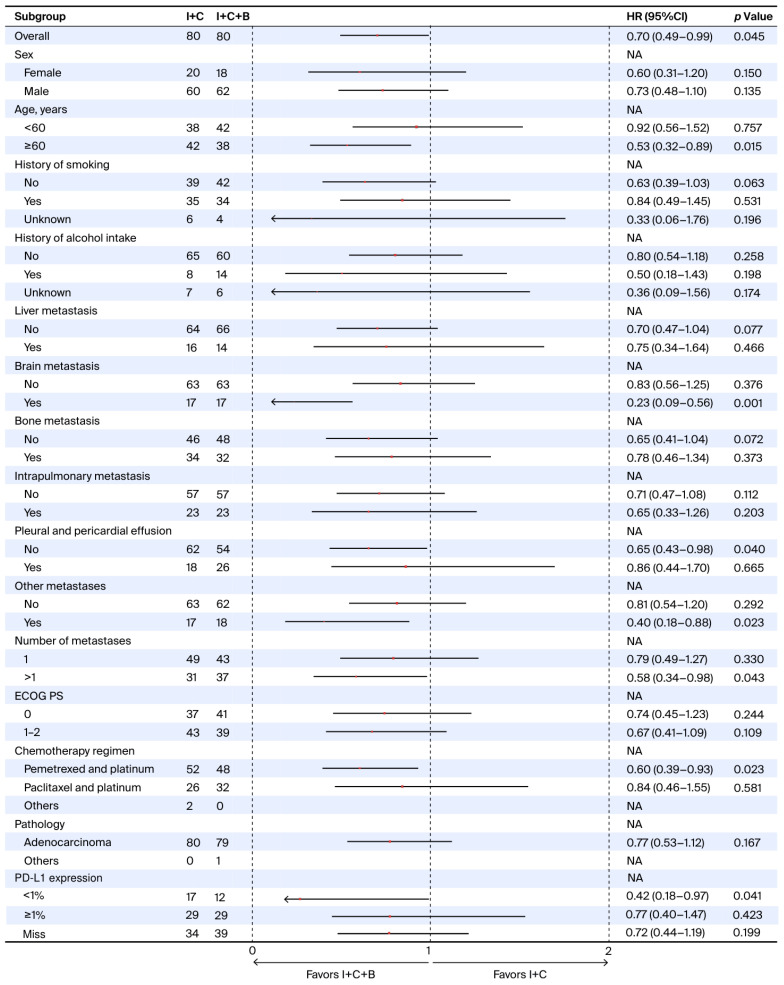
**Forest plot of progression-free survival subgroup analyses comparing I + C + B versus I + C.** Abbreviations: I + C + B, immune checkpoint inhibitors plus chemotherapy plus bevacizumab; I + C, immune checkpoint inhibitors plus chemotherapy; ECOG PS, Eastern Cooperative Oncology Group performance status; HR, hazard ratio; CI, confidence interval; NA, not available; PD-L1, programmed death-ligand 1.

Subgroup analyses were conducted on the post-PSM population to explore the differential effect of treatment across clinically relevant categories. Among patients aged ≥60 years, treatment with I + C + B was associated with significantly prolonged PFS compared to I + C (HR = 0.53; 95% CI: 0.32–0.89; *p* = 0.015). Similar benefits in PFS were observed in patients with brain metastases (HR = 0.23; 95% CI: 0.09–0.56; *p* = 0.001), those without pleural or pericardial effusions (HR = 0.65; 95% CI: 0.43–0.98; *p* = 0.040), patients with other metastatic sites (HR = 0.40; 95% CI: 0.18–0.88; *p* = 0.023), individuals presenting with more than one site of metastasis (HR = 0.58; 95% CI: 0.34–0.98; *p* = 0.043), and those receiving pemetrexed plus platinum (HR = 0.60; 95% CI: 0.39–0.93; *p* = 0.023). Notably, in the PD-L1 expression subgroups, I + C + B significantly improved PFS in patients with PD-L1 < 1% (HR = 0.42; 95% CI: 0.18–0.97; *p* = 0.041), whereas no significant PFS benefit was observed in the PD-L1 ≥ 1% subgroup (HR = 0.77; 95% CI: 0.40–1.47; *p* = 0.423) (Figure 2).

**Figure 3:**
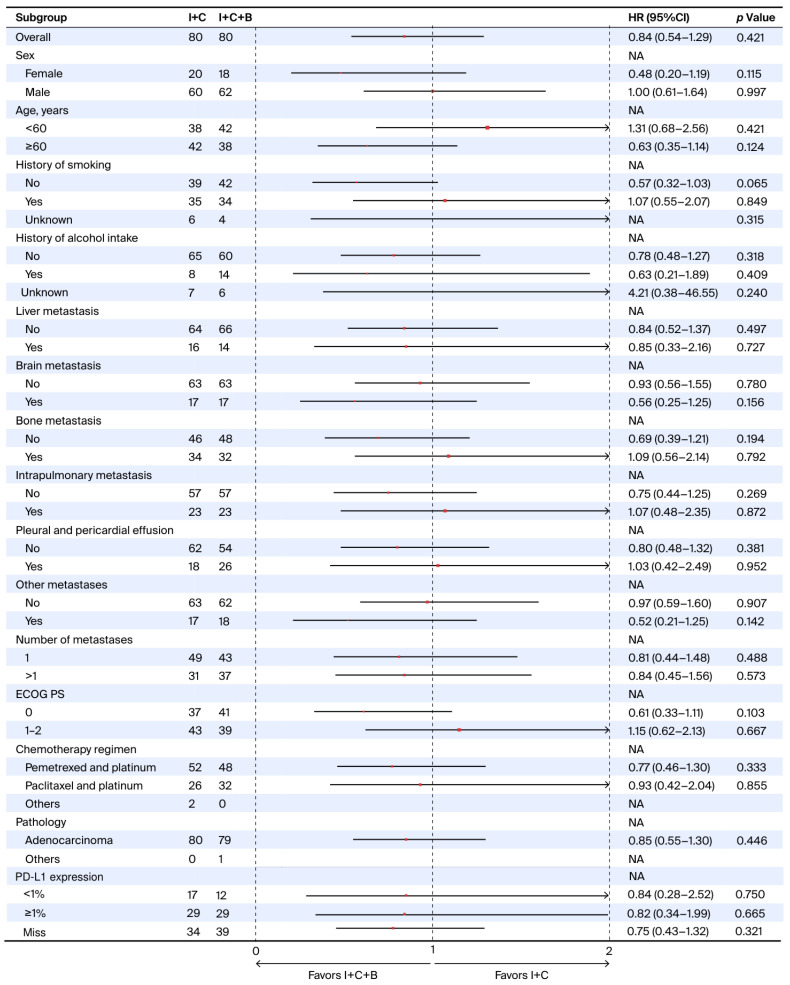
**Forest plot of overall survival subgroup analyses comparing I + C + B versus I + C.** Abbreviations: I + C + B, immune checkpoint inhibitors plus chemotherapy plus bevacizumab; I + C, immune checkpoint inhibitors plus chemotherapy; ECOG PS, Eastern Cooperative Oncology Group performance status; HR, hazard ratio; CI, confidence interval; NA, not available; PD-L1, programmed death-ligand 1.

Regarding OS, no statistically significant differences were observed across any analyzed subgroups (Figure 3). Specifically, I + C + B did not demonstrate a survival advantage over I + C regardless of PD-L1 status, including the PD-L1 < 1% subgroup (HR = 0.84; 95% CI: 0.28–2.52; *p* = 0.750) and the PD-L1 ≥ 1% subgroup (HR = 0.82; 95% CI: 0.34–1.99; *p* = 0.665).

### 3.4. Characteristics of Included Clinical Trials of Meta-Analysis

The literature search initially yielded 231 records. After title and abstract screening, followed by full-text assessment, four trials comprising a total of 2026 patients with non-squamous NSCLC were included in the final meta-analysis [16,19,25,26] (Figure S2). The Cochrane risk of bias assessment revealed that three studies were judged to have a high risk in the domain of blinding of participants and personnel due to their open-label design. No other domains were judged to have significant risk of bias in the included trials (Figure S3). Trial characteristics and the network evidence structure are summarized in Table 2 and Figure S4, respectively.

### 3.5. Meta-Analysis of Survival and Tumor Response

**Figure 4:**
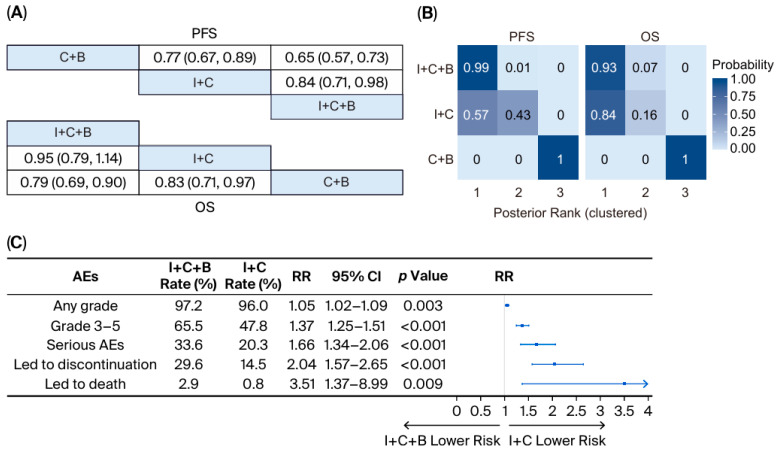
**Meta-analysis of survival outcomes and adverse events comparing I + C + B versus I + C.** (**A**) Network meta-analysis of PFS and OS; (**B**) posterior rank probabilities for each intervention; (**C**) forest plot of AEs from clinical trials. Abbreviations: I + C + B, immune checkpoint inhibitors plus chemotherapy plus bevacizumab; I + C, immune checkpoint inhibitors plus chemotherapy; C + B, chemotherapy plus bevacizumab; PFS, progression-free survival; OS, overall survival; AEs, adverse events; RR, risk ratio; CI, confidence interval. Notes: Regarding the rank probabilities, ranks are displayed on the ordinal scale (1 = best). Interventions are grouped into rank clusters representing non-exclusive superiority sets; multiple interventions within the same cluster may share identical posterior ranks.

The network meta-analysis demonstrated that I + C + B was associated with improved PFS compared to I + C (HR = 0.84; 95% CI: 0.71–0.98), while OS was comparable between groups (HR = 0.95; 95% CI: 0.79–1.14) (Figure 4A). Node-splitting analysis demonstrated no significant inconsistency between direct and indirect evidence (*p* = 0.610). Rank-cluster analysis revealed that for PFS, I + C + B uniquely occupied the superior tier (MNBT: 0 [95% CI: 0–0]) while I + C followed in the intermediate tier (MNBT: 0 [0, 1]); for OS, both regimens demonstrated comparable clinical priority within the top-performing tier (MNBT: 0 for both) (Figure 4B).

To facilitate an intuitive comparison, the median PFS and OS values across the included trials were pooled by treatment group. Among patients receiving I + C + B, the pooled median PFS was 9.27 months (95% CI: 8.47–10.07), compared to 6.33 months (95% CI: 5.63–7.03) for those receiving I + C. The corresponding median OS were 25.93 months (95% CI: 19.07–32.79) and 22.69 months (95% CI: 14.97–30.42), respectively.

### 3.6. Toxicity

In the whole real-world cohort, AEs of any grade were reported in 98.8% of patients receiving I + C + B compared with 96.4% in the I + C group (RR = 1.03; 95% CI: 0.99–1.07; *p* = 0.220). Grade 3–5 AEs occurred in 55.7% of the I + C + B group versus 40.0% in the I + C group (RR = 1.39; 95% CI: 1.07–1.82; *p* = 0.015) (Figure 1G). Notably, bevacizumab-associated toxicities were also more frequent in the I + C + B arm, including hypertension (any grade: 9.0% vs. 6.4%), proteinuria (any grade: 5.4% vs. 1.8%), and upper gastrointestinal hemorrhage (any grade: 2.4% vs. 0.0%). One fatal case of massive gastric bleeding was documented in the I + C + B group (Table S1).

Consistent with these findings, our meta-analysis showed that both the incidence and severity of AEs were elevated in the I + C + B group across all evaluated categories. Specifically, risks were significantly higher for any-grade AEs (RR = 1.05; 95% CI: 1.02–1.09; *p* = 0.003), grade 3–5 AEs (RR = 1.37; 95% CI: 1.25–1.51; *p* < 0.001), serious AEs (RR = 1.66; 95% CI: 1.34–2.06; *p* < 0.001), AEs leading to treatment discontinuation (RR = 2.04; 95% CI: 1.57–2.65; *p* < 0.001), and fatal AEs (RR = 3.51; 95% CI: 1.37–8.99; *p* = 0.009) (Figure 4C).

## 4. Discussion

In this real-world retrospective study, we demonstrated that the addition of bevacizumab to first-line I + C significantly improved PFS compared to I + C alone in patients with advanced non-squamous NSCLC without EGFR and ALK alterations. While OS was comparable, the results suggest that bevacizumab enhances short-term disease control but does not extend long-term survival. Notably, certain patient subgroups—particularly PD-L1-negative, the elderly patients and those with baseline brain metastases—appeared to derive greater PFS benefit from the I + C + B. To contextualize these findings, we performed a meta-analysis of previous studies: this confirmed that while I + C + B offers a clear PFS advantage, it does not translate into a significant OS improvement over the I + C regimen. However, the addition of bevacizumab was associated with increased treatment-related toxicity, reflecting the known risk profile of anti-angiogenic therapy [27,28]. These results underscore a key clinical dilemma—balancing the short-term efficacy gains against markedly added toxicity. Consequently, our data suggest that there is no clear clinical subgroup where the overall benefit definitively outweighs these safety risks.

The addition of bevacizumab improved median PFS, suggesting that dual inhibition of VEGF-driven angiogenesis and immune checkpoints initially yields more durable tumor control. Biologically, bevacizumab may normalize tumor vasculature and mitigate VEGF-mediated immunosuppression, thereby enhancing the delivery of chemotherapy and infiltration of immune effector cells into the tumor microenvironment [29,30,31]. Network meta-analyses have identified the atezolizumab–bevacizumab–chemotherapy combination as providing the greatest PFS among first-line regimens [32]. However, our analysis showed that this PFS advantage was transient: while the 1-year PFS rate was significantly higher in the I + C + B group (50.2% vs. 31.0%, *p* = 0.013), the 3-year PFS rates converged (12.5% vs. 12.9%, *p* = 0.958). Consequently, I + C + B did not demonstrate a statistically significant OS advantage (HR = 0.84, *p* = 0.421), with comparable 3-year OS rates (46.8% vs. 39.4%, *p* = 0.390). This finding suggests that the early disease control conferred by bevacizumab may not necessarily extend long-term survival once subsequent therapies and tumor biology take effect. One explanation is that while anti-angiogenic therapy initially normalizes vasculature, prolonged use may induce intra-tumoral hypoxia and trigger compensatory pathways, leading to acquired resistance. Furthermore, the potential survival benefit might be diluted by the “catch-up” effect, as patients receiving I + C alone may receive salvage therapies (including bevacizumab) upon progression, thereby narrowing the survival gap over time. Additionally, the modest sample size and relatively short follow-up may have limited this study’s ability to detect an OS difference. Importantly, our observation aligns with both our findings and a recently published report [30]. Similarly, earlier trials from the pre-immunotherapy era noted that adding bevacizumab to chemotherapy improved PFS without a commensurate OS gain in many cases [33,34]. These data underscore that OS is influenced by many factors beyond initial regimen efficacy, including tumor genetics, post-progression treatments, and patient comorbidities. Taken together, these findings indicate that while bevacizumab intensification enhances short-term tumor control, it fails to improve ultimate survival in the context of modern I + C, highlighting the substantial difficulty in identifying which patients, if any, could attain a meaningful survival benefit from I + C + B.

Our subgroup analyses provide insight into which patients might preferentially benefit from bevacizumab augmentation. We found that the PFS benefit of I + C + B was more pronounced in patients with PD-L1-negative expression (HR = 0.42, *p* = 0.041), while no significant difference was observed in the PD-L1-positive subgroup (*p* = 0.423). From a mechanistic perspective, bevacizumab may help overcome an immunosuppressive tumor microenvironment in PD-L1-negative tumors, potentially sensitizing “immune-cold” tumors to immunotherapy [35,36]. For example, co-mutations in genes such as STK11 and KEAP1 are known to confer resistance to immunotherapy by promoting an immune-cold microenvironment; encouragingly, the IMpower150 subgroup analysis showed that patients with KRAS-mutant tumors harboring STK11/KEAP1 mutations had improved survival with I + C + B [37]. This suggests that anti-angiogenic therapy might partially offset unfavorable biology in certain subsets. However, it must be emphasized that these PFS gains did not translate into an OS benefit.

Regarding other clinical strata, elderly patients (age ≥ 60) showed a notably larger PFS gain from the triplet regimen in our cohort. This result is intriguing, as older patients are often underrepresented in clinical trials and can have attenuated immune function or less tolerance for aggressive therapy. It has been reported that immunotherapy efficacy is largely preserved in fit older adults [38], and our findings suggest that adding bevacizumab is feasible and effective in this group when careful patient selection is applied. The combination of an immune checkpoint inhibitor with an anti-VEGF agent may counteract age-related immunosuppressive tumor environments, potentially explaining the enhanced PFS in our older subset. Similarly, patients with baseline brain metastases derived especially significant benefit from the addition of bevacizumab in our study. This differential effect can be explained by bevacizumab’s mechanism of action in the context of brain metastases. Bevacizumab can reduce tumor-associated vasogenic edema and normalize the blood–brain barrier to some extent, potentially enhancing drug delivery and controlling microscopic metastatic growth [39,40]. Indeed, real-world analyses have suggested that bevacizumab may confer a greater survival benefit in NSCLC patients with brain metastases than in those without [29,41].

Despite these observations in specific clinical scenarios, it is important to note that the PFS advantages across all subgroups—including age, brain metastases, and PD-L1 status—were not accompanied by statistically significant improvements in OS. Given that the clinical benefit is marginal relative to the potential for severe adverse events, our findings do not support I + C + B as a recommended standard for the general population. If this intensification is considered, it should be restricted to highly selected patients with a robust performance status and minimal bleeding risk. Furthermore, such use necessitates rigorous monitoring for anti-angiogenic-related toxicities and a thorough discussion of the unfavorable risk–benefit ratio with the patient.

The safety profile of the I + C + B regimen in our study aligned with the expected risks of anti-angiogenic therapy. Consistent with our meta-analysis and the IMpower150 trial [42,43,44], the addition of bevacizumab did not introduce novel safety signals but significantly increased the incidence of treatment-related AEs, particularly grade ≥3 hypertension and hematologic toxicities. While bevacizumab improves tumor control, the associated risks must be prioritized over the modest PFS gains given the absence of a clear survival advantage. For instance, a patient with brain metastases achieving stability on I + C + B therapy might avoid neurological deterioration that would occur with early progression. Nonetheless, the significantly elevated risk of fatal AEs (RR = 3.51) identified in our meta-analysis strongly suggests that the added toxicity may not be justified for the majority of patients. Oncologists must weigh these factors on an individual basis. In summary, from a safety standpoint, bevacizumab adds significant and potentially life-threatening toxicity to first-line therapy, which must be judiciously weighed against the limited short-term clinical benefit on a strictly case-by-case basis.

Notwithstanding these findings, several limitations warrant consideration. First, the retrospective, non-randomized design introduces potential selection bias. While PSM was used to balance measurable baseline characteristics, it cannot account for the inherent prognostic differences between groups, particularly the more favorable eligibility profile of the I + C + B cohort due to stricter exclusion criteria (e.g., central, bleeding and necrotic tumors). Second, AEs were collected retrospectively and may have been incomplete due to underreporting in clinical records, though the observed safety profile aligned with meta-analysis findings. Retrospective collection also precluded robust analysis of quality of life due to the lack of systematic prospective assessments. Third, although we attempted to evaluate PD-L1 expression, data were unavailable for 45.8% of patients due to insufficient tissue or lack of baseline sampling. Since PD-L1 is a critical biomarker for predicting immunotherapy efficacy, this limited sample size may reduce the statistical power to identify it as a definitive predictive biomarker for treatment outcomes [45,46]. Furthermore, the subgroup analyses in this study were exploratory rather than hypothesis-driven, and no correction for multiple testing was applied. Consequently, these findings should be interpreted with caution, as there is an inherent potential for an inflated Type I error rate. Finally, the modest cohort size may limit the detection of subtle survival differences between the two regimens. Future prospective studies with standardized AE monitoring and biomarker-driven patient selection are warranted to refine the therapeutic strategies for EGFR/ALK wild-type non-squamous NSCLC.

## 5. Conclusions

In this study, the PFS advantage observed with I + C + B was tempered by the significantly increased toxicity and a lack of OS improvement. Given that these incremental gains fail to justify the risk of severe AEs across all analyzed subgroups, I + C + B cannot be recommended for routine use. Any implementation warrants judicious patient selection and vigilant monitoring. Ultimately, these results highlight a critical clinical trade-off: balancing modest radiological gains against an increased therapy-related burden in the absence of a clear survival advantage.

## Figures and Tables

**Table 1:** Baseline demographic characteristics.

Characteristics	Before PSM			After PSM		
	I + C	I + C + B	*p* Value	I + C	I + C + B	*p* Value
	(*N* = 110)	(*N* = 167)		(*N* = 80)	(*N* = 80)	
**Sex**			**0.798**			**0.853**
Female	28 (25.5%)	39 (23.4%)		20 (25.0%)	18 (22.5%)	
Male	82 (74.6%)	128 (76.7%)		60 (75.0%)	62 (77.5%)	
**Age, years (Q1, Q3)**			**0.050**			**0.693**
	60 (54, 68)	58 (52, 65)		60 (54, 65)	59 (54, 66)	
**History of smoking**			**0.771**			**0.769**
No	54 (49.1%)	87 (52.1%)		39 (48.8%)	42 (52.5%)	
Yes	48 (43.6%)	71 (42.5%)		35 (43.8%)	34 (42.5%)	
Unknown	8 (7.3%)	9 (5.4%)		6 (7.5%)	4 (5.0%)	
**History of alcohol intake**			**0.922**			**0.384**
No	86 (78.2%)	129 (77.3%)		65 (81.3%)	60 (75.0%)	
Yes	15 (13.6%)	22 (13.2%)		8 (10.0%)	14 (17.5%)	
Unknown	9 (8.2%)	16 (9.6%)		7 (8.8%)	6 (7.5%)	
**Liver metastasis**			**0.993**			**0.839**
No	92 (83.6%)	141 (84.4%)		64 (80.0%)	66 (82.5%)	
Yes	18 (16.4%)	26 (15.6%)		16 (20.0%)	14 (17.5%)	
**Brain metastasis**			**0.096**			**1.000**
No	86 (78.2%)	114 (68.3%)		63 (78.8%)	63 (78.8%)	
Yes	24 (21.8%)	53 (31.7%)		17 (21.3%)	17 (21.3%)	
**Bone metastasis**			**0.807**			**0.872**
No	67 (60.9%)	98 (58.7%)		46 (57.5%)	48 (60.0%)	
Yes	43 (39.1%)	69 (41.3%)		34 (42.5%)	32 (40.0%)	
**Intrapulmonary metastasis**			**0.843**			**1.000**
No	77 (70.0%)	120 (71.9%)		57 (71.3%)	57 (71.3%)	
Yes	33 (30.0%)	47 (28.1%)		23 (28.8%)	23 (28.8%)	
Other metastases *			**0.962**			**1.000**
No	90 (81.8%)	135 (80.8%)		63 (78.8%)	62 (77.5%)	
Yes	20 (18.2%)	32 (19.2%)		17 (21.3%)	18 (22.5%)	
**Number of metastases**			**0.736**			**0.424**
1	65 (59.1%)	94 (56.3%)		49 (61.3%)	43 (53.8%)	
>1	45 (40.9%)	73 (43.7%)		31 (38.8%)	37 (46.3%)	
**Pleural and pericardial effusion**			**0.209**			**0.215**
No	76 (69.1%)	128 (76.7%)		62 (77.5%)	54 (67.5%)	
Yes	34 (30.9%)	39 (23.4%)		18 (22.5%)	26 (32.5%)	
**ECOG performance status**			**0.298**			**0.635**
0	47 (42.7%)	82 (49.1%)		37 (46.3%)	41 (51.3%)	
1~2	63 (57.3%)	85 (50.9%)		43 (53.8%)	39 (48.8%)	
**Chemotherapy regimen**			**<0.001**			**0.249**
Pemetrexed and platinum ^†^	82 (74.6%)	71 (42.5%)		52 (65.0%)	48 (60.0%)	
Paclitaxel and platinum	26 (23.6%)	96 (57.5%)		26 (32.5%)	32 (40.0%)	
Others	2 (1.8%)	0 (0.0%)		2 (2.5%)	0 (0.0%)	
**Pathology**			**0.412**			**1.000**
Adenocarcinoma	110 (100.0%)	164 (98.2%)		80 (100.0%)	79 (98.8%)	
Others ^‡^	0 (0.0%)	3 (1.8%)		0 (0.0%)	1 (1.3%)	
**PD-L1 expression** ^※^			**0.450**			**0.548**
<1%	24(21.8%)	28(16.8%)		17(21.3%)	12(15.0%)	
≥1%	40(36.4%)	58(34.7%)		29(36.3%)	29(36.3%)	
Miss	46(41.8%)	81(48.5%)		34(42.5%)	39(48.8%)	

Abbreviations: PSM, propensity score matching; I + C, immune checkpoint inhibitor plus chemotherapy; I + C + B, immune checkpoint inhibitor plus chemotherapy and bevacizumab; ECOG, Eastern Cooperative Oncology Group; PD-L1, programmed death-ligand 1. * Metastases other than liver, brain, bone, and lung metastases. ^†^ Platinum: Including cisplatin, carboplatin, nedaplatin; Others: One patient for gemcitabine and cisplatin, and another for etoposide and carboplatin. ^‡^ Undifferentiated carcinoma. ^※^ Tumor proportion score.

**Table 2:** **The baseline table of articles included in the network meta-analysis.**

Study	Year	Phase	Treatment Strategies	Sample Size	Primary Endpoint	EGFR/ALK Wild-Type Subgroup PFS HR (95% CI)	EGFR/ALK Wild-Type Subgroup OS HR (95% CI)	EGFR/ALK Wild-Type Subgroup Median PFS (95% CI)	EGFR/ALK Wild-Type Subgroup Median OS HR (95% CI)	Any Grade AEs	Grade 3–5 AEs
APPLE	2024	III	I + C + B	143	PFS	0.97 (0.75–1.25)	0.99 (0.71–1.38)	9.3 (7.7–11.6)	28.0 (23.1–32.4)	99.0%	55.1%
			I + C	144				9.5 (7.6–23.8)	26.9 (22.0-NR)	99.5%	56.6%
ONO-4538-52	2023	III	I + C + B	275	OS	0.56 (0.43–0.71)	0.74 (0.58–0.94)	12.1 (9.8–14.0)	30.8 (26.8–34.7)	98.5%	74.7%
			C + B	275				NA	NA	NA	NA
IMpower151	2023	III	I + C + B	71	PFS	0.81 (0.55–1.19)	NA	10.4 (7.6–13.3)	NA	99.3%	74.3%
			C + B	71				NA	NA	NA	NA
IMpower150	2019	III	I + C + B	359	PFS, OS, Safety	0.57 (0.48–0.67) *	0.80 (0.67–0.95) *	8.3 (7.7–9.8)	19.5 (17.0–22.2)	98.2%	71.5%
			I + C	350		0.82 (0.70–0.97) *	0.84 (0.71–1.00) *	6.3 (5.6–7.0)	19.0 (15.7–21.5)	97.8%	63.1%
			C + B	338				NA	NA	NA	NA

Abbreviations: PFS, progression-free survival; OS, overall survival; HR, hazard ratio; 95% CI, 95% confidence interval; I + C + B, immune checkpoint inhibitors plus chemotherapy and bevacizumab; I + C, immune checkpoint inhibitors plus chemotherapy; C + B, chemotherapy plus bevacizumab; AEs, treatment-related adverse events; NA, not available. * Compared with C + B.

## Data Availability

The data presented in this study are available on request from the corresponding author due to ethical reasons.

## Supplementary Materials

The following supporting information can be downloaded at https://www.mdpi.com/article/10.3390/curroncol33030173/s1, Figure S1: Retrospective study flow chart of patient selection for I + C + B and I + C treatment groups; Figure S2: Flow diagram of literature retrieval and selection; Figure S3: Quality assessment of included studies using Cochrane Collaboration’s tool; Figure S4. Network Plot of the Direct Comparisons among Treatment Regimens; Table S1: Treatment-related AEs of the real-world cohort; File S1: Supplementary methods: Search strategies for retrieving papers in each database; File S2: PRISMA-NMA checklist of items to include when reporting a systematic review involving a network meta-analysis.

## Abbreviations

The following abbreviations are used in this manuscript:

NSCLC	Non-Small Cell Lung Carcinoma
EGFR	Epidermal Growth Factor Receptor
ALK	Anaplastic Lymphoma Kinase
I + C	Immune Checkpoint Inhibitors combined with Chemotherapy
PFS	Progression-Free Survival
HR	Hazard Ratio
ORR	Objective Response Rate
OS	Overall Survival
PD-L1	Programmed Death-Ligand 1
VEGF	Vascular Endothelial Growth Factor
I + C + B	Immune Checkpoint Inhibitors plus Chemotherapy and Bevacizumab
AEs	Adverse Events
RR	Relative Risk
CIs	Confidence Intervals
PSM	Propensity Score Matching
SMDs	Standardized Mean Differences
SD	Stable Disease
CR	Complete Response
PR	Partial Response
PD	Progressive Disease
RECIST	Response Evaluation Criteria in Solid Tumors
CNS	Central Nervous System
SEER	Surveillance Epidemiology and End Results
