# Supplementary material for: Comparative Efficacy and Safety of First-Line Immune Checkpoint Inhibitors Plus Chemotherapy with or Without Bevacizumab in Advanced Non-Squamous Non-Small Cell Lung Carcinoma
Source: Curr Oncol. 2026 Mar 18;33(3):173. doi: 10.3390/curroncol33030173 (PMC13025701; doi:10.3390/curroncol33030173)

Figure S1. Retrospective Study Flow Chart of Patient Selection for I+C+B and I+C Treatment Groups.

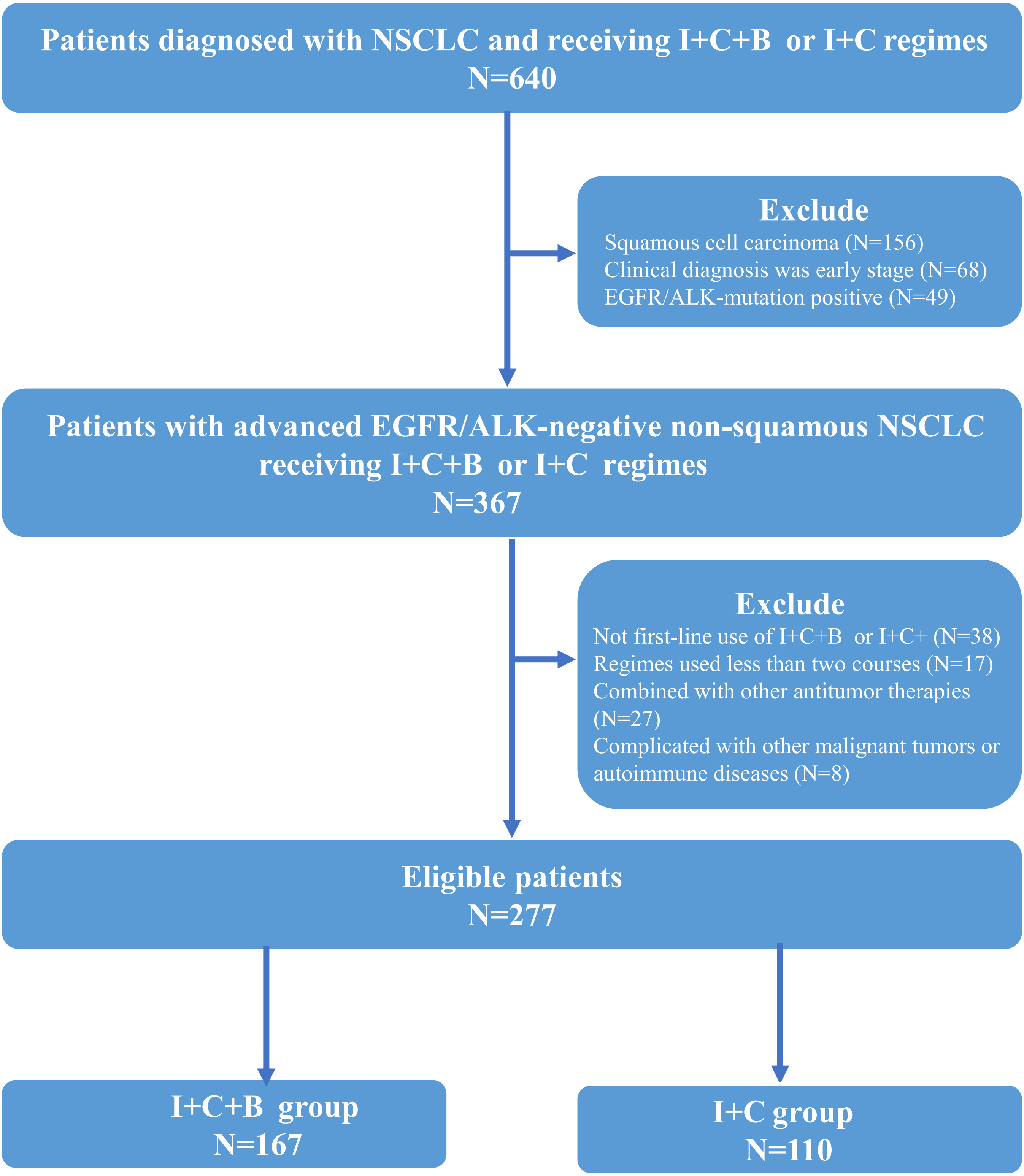

Supplement: Supplementary file 1 [file curroncol-33-00173-s001.zip › Figure S1.pdf]
