# Supplementary material for: Comparative Efficacy and Safety of First-Line Immune Checkpoint Inhibitors Plus Chemotherapy with or Without Bevacizumab in Advanced Non-Squamous Non-Small Cell Lung Carcinoma
Source: Curr Oncol. 2026 Mar 18;33(3):173. doi: 10.3390/curroncol33030173 (PMC13025701; doi:10.3390/curroncol33030173)
Supplement: Supplementary file 1 [file curroncol-33-00173-s001.zip › File S1.pdf]

File S1: Supplementary methods: Search strategies for retrieving papers in each database.

Pubmed

("Bevacizumab" [MeSH Terms] OR "Bevacizumab" [Title/Abstract] OR "Mvasi" [Title/Abstract] OR "Bevacizumab-awwb" [Title/Abstract] OR "Bevacizumab awwb" [Title/Abstract] OR "Avastin" [Title/Abstract] OR “Zirabev” [Title/Abstract] OR “QL1101” [Title/Abstract] OR “IBI305” [Title/Abstract] OR “LY01008” [Title/Abstract] OR “MIL60” [Title/Abstract] OR “TAB008” [Title/Abstract] OR “HLX04” [Title/Abstract]) AND

("Chemotherapy" [Title/Abstract] OR "Chemotherapies" [Title/Abstract] OR "Cisplatin" [Title/Abstract] OR "Carboplatin" [Title/Abstract] OR “Platinum” [Title/Abstract] OR "Paraplatin" [Title/Abstract] OR "Paraplatine" [Title/Abstract] OR "Ribocarbo" [Title/Abstract] OR "Carboplat" [Title/Abstract] OR "Ercar" [Title/Abstract] OR "Pemetrexed" [Title/Abstract] OR "Paclitaxel" [Title/Abstract]) AND

("pembrolizumab" [Supplementary Concept] OR "pembrolizumab" [Title/Abstract] OR "lambrolizumab" [Title/Abstract] OR "Keytruda" [Title/Abstract] OR "MK-3475" [Title/Abstract] OR "nivolumab" [Supplementary Concept] OR "nivolumab" [Title/Abstract] OR "MDX-1106" [Title/Abstract] OR "ONO-4538" [Title/Abstract] OR "BMS-936558" [Title/Abstract] OR "Opdivo" [Title/Abstract] OR "atezolizumab" [Title/Abstract] OR "MPDL3280A" [Title/Abstract] OR "Tecentriq" [Title/Abstract] OR "RG7446" [Title/Abstract] OR "RG-7446" [Title/Abstract] OR "Durvalumab" [Title/Abstract] OR "Imfinzi" [Title/Abstract] OR "MEDI4736" [Title/Abstract] OR "Camrelizumab" [Title/Abstract] OR "SHR-1210" [Title/Abstract] OR "Tislelizumab" [Title/Abstract] OR "Sintilimab" [Title/Abstract] OR "anti-PDL1" [Title/Abstract] OR "anti-PD1" [Title/Abstract] OR "PD-1" [Title/Abstract] OR "PD-L1" [Title/Abstract] OR "Programmed Death 1"[Title/Abstract] OR "Programmed Cell Death 1 Receptor" [Title/Abstract] OR "Programmed Death Ligand 1" [Title/Abstract] OR "Programmed Cell death 1 Ligand 1 protein" [Title/Abstract] OR "immune checkpoint inhibitor" [Title/Abstract] OR "immune therapy" [Title/Abstract] OR "immunotherapy" [Title/Abstract]) AND

(“Carcinoma, Non-Small-Cell Lung” [MeSH Terms] OR "Carcinoma, Non Small Cell Lung" [Title/Abstract] OR "Carcinomas, Non-Small-Cell Lung" [Title/Abstract] OR "Lung Carcinoma, Non-Small-Cell" [Title/Abstract] OR "Lung Carcinomas, Non-Small-Cell" [Title/Abstract] OR "Non-Small-Cell Lung Carcinomas" [Title/Abstract] OR "Non-Small-Cell Lung Carcinoma" [Title/Abstract] OR "Non Small Cell Lung Carcinoma" [Title/Abstract] OR "Carcinoma, Non-Small Cell Lung" [Title/Abstract] OR "Non-Small Cell Lung Carcinoma" [Title/Abstract] OR "Non-Small Cell Lung Cancer" [Title/Abstract] OR "Nonsmall Cell Lung Cancer" [Title/Abstract]) AND

(“randomized controlled trial” [Title/Abstract] OR “controlled clinical trial” [Title/Abstract] OR “clinical trials” [Title/Abstract] OR “trial” [Title/Abstract] OR random* [Title/Abstract] OR placebo* [Title/Abstract] OR “phase” [Title/Abstract])

Embase

('bevacizumab'/exp OR 'bevacizumab' OR 'bevacizumab':ab,ti OR 'mvasi':ab,ti OR 'bevacizumab-awwb':ab,ti OR 'bevacizumab awwb':ab,ti OR 'avastin':ab,ti OR 'zirabev':ab,ti OR 'ql1101':ab,ti OR 'ibi305':ab,ti OR 'ly01008':ab,ti OR 'mil60':ab,ti OR 'tab008':ab,ti OR 'hlx04':ab,ti) AND ('chemotherapy':ab,ti OR 'chemotherapies':ab,ti OR 'cisplatin':ab,ti OR 'carboplatin':ab,ti OR 'paraplatin':ab,ti OR 'paraplatine':ab,ti OR 'ribocarbo':ab,ti OR 'carboplat':ab,ti OR 'ercar':ab,ti OR 'pemetrexed':ab,ti OR 'paclitaxel':ab,ti) AND ('pembrolizumab'/exp OR 'pembrolizumab' OR 'pembrolizumab':ab,ti OR 'lambrolizumab':ab,ti OR 'keytruda':ab,ti OR 'mk-3475':ab,ti OR 'nivolumab':ab,ti OR 'mdx-1106':ab,ti OR 'ono-4538':ab,ti OR 'bms-936558':ab,ti OR 'opdivo':ab,ti OR 'atezolizumab':ab,ti OR 'mpdl3280a':ab,ti OR 'tecentriq':ab,ti OR 'rg7446':ab,ti OR 'rg-7446':ab,ti OR 'durvalumab':ab,ti OR 'imfinzi':ab,ti OR 'medi4736':ab,ti OR 'camrelizumab':ab,ti OR 'shr-1210':ab,ti OR 'tislelizumab':ab,ti OR 'sintilimab':ab,ti OR 'anti-pdl1':ab,ti OR 'anti-pd1':ab,ti OR 'pd-1':ab,ti OR 'pd-l1':ab,ti OR 'programmed death 1':ab,ti OR 'programmed cell death 1 receptor':ab,ti OR 'programmed death ligand 1':ab,ti OR 'programmed cell death 1 ligand 1 protein':ab,ti OR 'immune checkpoint inhibitor':ab,ti OR 'immune therapy':ab,ti OR 'immunotherapy':ab,ti) AND ('carcinoma, non small cell lung'/exp OR 'carcinomas, non-small-cell lung':ab,ti OR 'lung carcinoma, non-small-cell':ab,ti OR 'lung carcinomas, non-small-cell':ab,ti OR 'non-small-cell lung carcinomas':ab,ti OR 'non-small-cell lung carcinoma':ab,ti OR 'non small cell lung carcinoma':ab,ti OR 'carcinoma, non-small cell lung':ab,ti OR 'non-small cell lung carcinoma':ab,ti OR 'non-small cell lung cancer':ab,ti OR 'nonsmall cell lung cancer':ab,ti) AND ('crossover procedure':de OR 'double-blind procedure':de OR 'randomized controlled trial':de OR 'single-blind procedure':de OR (random* OR factorial* OR crossover* OR cross NEXT/1 over* OR placebo* OR doubl* NEAR/1 blind* OR singl* NEAR/1 blind* OR assign* OR allocat* OR volunteer*):de,ab,ti)

Cochrane

#1 MeSH descriptor: [Bevacizumab] explode all trees 3053

#2 Chemotherapy OR Chemotherapies OR Cisplatin OR Carboplatin OR Paraplatin OR Paraplatine OR Ribocarbo OR Carboplat OR Ercar OR Pemetrexed OR Paclitaxel 110257

#3 MeSH descriptor: [Carcinoma, Non-Small-Cell Lung] explode all trees 6577

#4 pembrolizumab OR pembrolizumab OR lambrolizumab OR Keytruda OR MK-3475 OR nivolumab OR nivolumab OR MDX-1106 OR ONO-4538 OR BMS-936558 OR Opdivo OR atezolizumab OR MPDL3280A OR Tecentriq OR RG7446 OR RG-7446 OR Durvalumab OR Imfinzi OR MEDI4736 OR Camrelizumab OR SHR-1210 OR Tislelizumab OR Sintilimab OR anti-PDL1 OR anti-PD1 OR PD-1 OR PD-L1 OR Programmed Death 1 OR Programmed Cell Death 1 Receptor OR Programmed Death Ligand 1 OR Programmed Cell death 1 Ligand 1 protein OR immune checkpoint inhibitor OR immune therapy OR immunotherapy 43715

#5 randomized controlled trial OR controlled clinical trial OR clinical trials OR trial OR random* OR placebo* OR phase 1862265

#6 #1 AND #2 AND #3 AND #4 AND #5 45
